# Splenic Abscess due to Brucella Melitensis - A Rare Pediatric Complication

**DOI:** 10.4103/0974-2727.72212

**Published:** 2010

**Authors:** Aisha M Parande, B G Mantur, Mahesh Kore, Eranna Palled

**Affiliations:** Department of Microbiology, Belgaum Institute of Medical Sciences, Belgaum, Karnataka, India; 1 Shree Pediatric Hospital and Neonatal Care Center, Nipani, Belgaum, Karnataka, India; 2 Department of Radiology, Belgaum Institute of Medical Sciences, Belgaum, Karnataka, India

**Keywords:** Acute brucellosis, serology, splenic abscess

## Abstract

Splenic abscess due to Brucella species is an extremely rare complication especially in acute illness. Here we report a case of splenic abscess caused by *Brucella melitensis* biotype 1 in a child with acute infection who was successfully treated with only antibiotics.

## INTRODUCTION

Brucella species frequently associated with human brucellosis are *Brucella melitensis*, *B. abortus* and *B. suis*. Human brucellosis, a zoonosis, is a multisystem disease that may present with a wide spectrum of clinical manifestations. Brucellosis is known for its complications, of which gastrointestinal brucellosis is uncommon. The most common clinical forms of gastrointestinal brucellosis reported, include chronic liver disease, acute cholecystitis, ascites, acute ileitis, ulcerative colitis and pancreatitis.[1] Splenic abscess is an extremely rare but serious complication of brucellosis, more so of chronic brucellosis. Very few cases of splenic abscess in brucellosis are described in the literature, most of them in adults.[2–9] Of these, only two cases documented are in the pediatric age group.[5,6] Here we report a case of splenic abscess due to *B. melitensis* in a child with acute brucellosis.

## CASE REPORT

An eight-year-old male child was referred to our laboratory from Dr. Kore’s Shree pediatric hospital, Nipani for microbiological evaluation of brucellosis. He had history of intermittent fever and occasional abdominal pain of 45 days duration. He also complained of pain in the right shoulder. There was history of decreased appetite and weight loss. The patient gave a history of consumption of raw goat’s milk. The patient’s father was a farmer by occupation. There was no other significant family history. The patient was previously being treated by a general practitioner with antibiotics to which he did not respond. The patient was apparently not given anti-brucellar therapy. Physical examination revealed an ill-looking child with temperature of 101°F. The spleen was palpable 2 cm below the costal margin and liver was just palpable. Abdominal guarding was present. There was no lymphadenopathy or signs of meningeal irritation.

**Figure 1:**
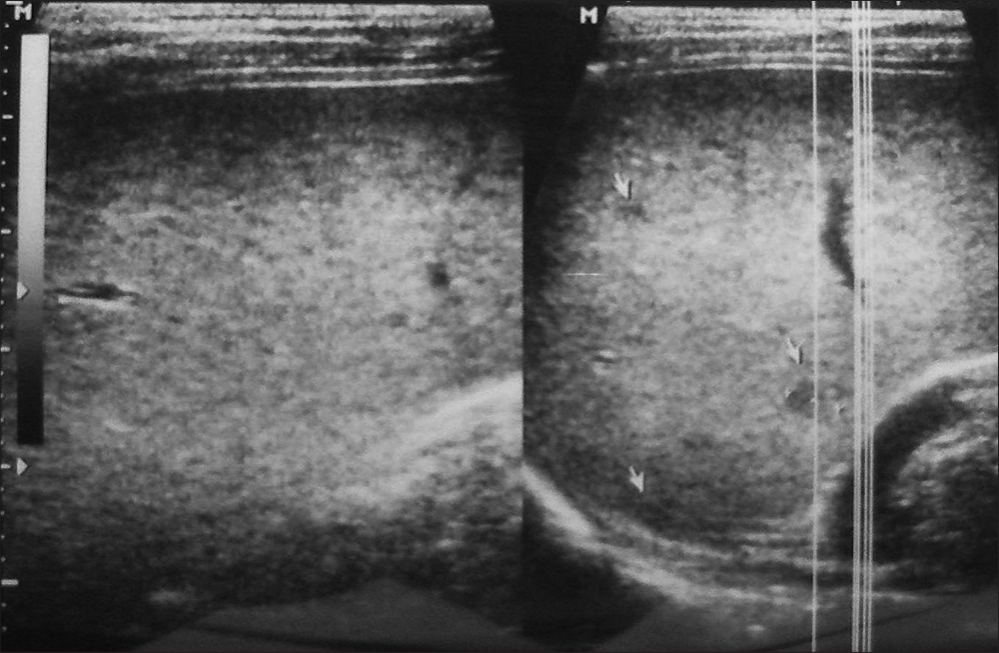
USG of spleen showing small echo-poor focal infiltrates (arrow)

The investigations showed an Hb of 8.7 gm%, total count of 6900/mm^3^, differential counts were within normal limits and the erythrocyte sedimentation rate was raised to 60 mm after one hour. Total and direct bilirubin levels were within normal limits. Test for HIV-1 and HIV-2, Widal test and peripheral blood smear for malaria parasites were negative. The Mantoux test was negative. The serum tested a week prior to presenting to this laboratory was reported negative for Brucella antibodies. In our laboratory, it tested positive by the Rose Bengal Plate agglutination Test (RBPT) and positive at a dilution of 1 : 640 by Standard Tube Agglutination test (SAT) and at a dilution of 1 : 20 by tube agglutination test using 2 mercaptoethanol (2ME). Antigens for the tests were obtained from the Indian Veterinary Research Institute (IVRI), Izatnagar, India. Ultrasonography (USG) of the abdomen revealed multiple 2–4 mm echo-poor focal splenic infiltrates [Figure 1]. The specific diagnosis of Brucellar splenic abscess was delayed for almost one and half months.

Blood was cultured on Brain Heart Infusion (BHI) biphasic media. Meanwhile, keeping in mind the diagnosis of brucellosis, a free-hand USG-guided diagnostic splenic aspiration was performed. Prior to this, it was ensured that the patient had acceptable coagulation parameters. The aspirated material was cultured aerobically and anaerobically on chocolate agar, Lowenstein-Jensen (LJ) medium, thioglycollate broth, Castaneda’s biphasic BHI medium and Sabouraud’s dextrose agar (SDA). The blood and splenic aspirate cultures yielded growth of *B. melitensis* on the 11^th^ and 7 ^th^ day, respectively. The isolate was confirmed as *B. melitensis* biotype 1 at IVRI Izatnagar, India. The SDA, thioglycollate and LJ media did not yield any growth after 30 days and eight weeks of incubation, respectively.

**Figure 2:**
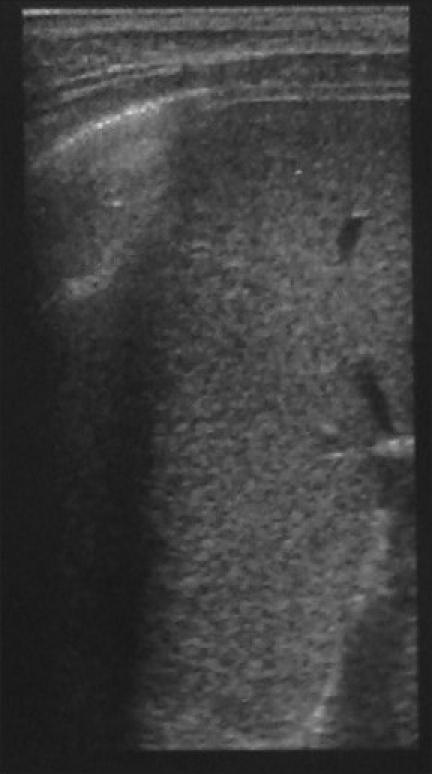
Normal splenic echotexture seen on follow-up USG

Following SAT and 2ME results, the child was started on oral doxycycline 4 mg/kg/day in two divided doses for six weeks together with injectable streptomycin 15 mg/kg/day once a day for initial two weeks. The child became afebrile and was discharged from the hospital on 10^th^ day of admission. On follow-up after two months, the child had gained appetite and weight. The serum titres of SAT had decreased to dilutions of 1 : 80 and RBPT and 2ME tests were negative. On repeat USG, the splenic abscesses had regressed in size and number. At the last follow-up after nine months, the serological tests for Brucella antibodies were negative and abdominal USG revealed spleen with normal echotexture [Figure 2].

## DISCUSSION

Splenic abscess formation is usually reported in chronic hepatosplenic brucellosis. Splenic abscess as a complication of acute brucellosis is considered exceptional with an incidence of 2–3% in the largest series.[3,10] A review of world literature has documented only two cases of splenic abscess in acute brucellosis in children.[5,6]

Inspite of the high prevalence of brucellosis in animals and favorable factors for transmission to human beings, the diagnosis of brucellosis can be easily missed due to lack of clinical suspicion by the healthcare provider and lack of appropriate diagnostic facilities. This delay in diagnosis could result in an increase in the incidence of sequelae. In our case too, these factors contributed to the initial delay in diagnosis as the disease was not suspected by the general practitioner whom the patient had first consulted. Also the serum test for brucellosis in the outside laboratory was negative. Prozone effect or the use of non-standardized antigens could be the possible explanations for a false-negative serological test. However, because of the epidemiological indication and strong clinical suspicion by the treating pediatrician, the brucella antibody tests were re-evaluated in our laboratory and found to be positive. Thus serological tests may require repeated evaluation over several weeks for accurate diagnosis especially when there is a high-degree of clinical suspicion.

In a study by Al-Eissa *et al*.[11] among the 102 consecutive cases of childhood brucellosis, 91% had fever, 35% splenomegaly, 28% hepatomegaly and 16% lymphadenopathy. Our patient also presented with fever, hepatosplenomegaly, anorexia, weight loss and arthralgia although lymphadenopathy was absent.

With the advent of noninvasive imaging techniques such as USG and computed tomography scan, localization and increased accuracy in aspiration of splenic material has increased diagnostic yield. In this case, the aspiration has yielded the material demonstrating the organisms.

The mortality rate of splenic abscess is reported to be 100% without treatment.[6] The prognosis of splenic abscess is good with early diagnosis and treatment, as in our case. Although it is reported that surgical drainage or splenectomy may be required in the treatment of splenic abscesses,[12] because of progressive clinical improvement of the patient and decreasing size of lesions, surgical intervention was not required in this case. Our patient responded with medical treatment alone. This could be attributed to the smaller size of the abscesses although multiple in number. Successful medical treatment of acute Brucella splenic abscess with antibiotics alone has been reported by others also.[2,4] Table 1 highlights the features of the reported cases in the literature of splenic abscess due to brucellosis.

**Table 1:** Features of the reported cases in the literature of splenic abscess due to brucellosis

Case no. (reference)	Patient’s age (y)/sex	Risk factor	Localization	Brucella antibody titre	Isolate (source)	Therapy	Outcome
1 (8)	51/M	Farmer	Liver/spleen, bone	1 : 320	*B.suis* (draining sinus from RUQ)	Surgical drainage + Tet	Recovered
2 (8)	58/M	Butcher	Spleen	1 : 320	*B. suis* (spleen)	Splenectomy + Tet × 3 m	Recovered
3 (8)	54/M	Cattle	buyer Liver/spleen, lymph node	1 : 80	*B.suis* (draining sinus from LUQ)	Not specified (NS)	Died of hemorrhage from esophageal varices
4 (8)	53/M	Farmer	Spleen	1 : 1280	*B. suis* (spleen)	Splenectomy + Tet	Recovered
5 (8)	53/M	Farmer	Spleen	1 : 1280	None	Splenectomy + antibiotic (NS)	Recovered
6 (5)	3/M	Unknown	Liver/spleen	1 : 1280	*B. melitensis* (Liver, bone marrow)	Percutaneous drainage of liver abscess + Rif and TMP-SMZ × 2 m	Recovered
7 (3)	53/M	Past history of brucellosis	Spleen	1 : 80	Negative	2 cycles of Doxy × 2 m and inj. Strepto × 21 d	Relapsed
8 (3)	80/NS	Past history of brucellosis	Spleen	1 : 320	Negative	Doxy × 2 m, inj. Strepto × 21 d + splenectomy followed by Doxy, Rif × 3 m	Died due to causes unrelated to brucellosis
9 (3)	72/NS	Past history of brucellosis	Spleen	1 : 40	Negative	Doxy × 2 m, inj. Strepto × 21 d + splenectomy followed by Doxy, Rif × 3 m	Recovered
10 (2)	70/F	Consumption of unpasteurized milk products	Spleen	1 : 320	*B. melitensis* (Blood)	Doxy, Rif × 6 weeks, inj. Strepto × 21 d	Recovered
11 (9)	19/M	Contact with dairy products	Spleen, aortic valve	High titres of SAT	*B. melitensis* (Blood, spleen, vegetations)	Splenectomy + antibiotics (NS)	Recovered
12 (4)	61/M	Unknown	Spleen	1 : 1280	Negative	Rif, Doxy and TMP-SMZ	Recovered
13 (7)	45/M	Livestock industry worker	Spleen, aortic valve	1 : 160	B. abortus (Blood)	Genta and Rif, Doxy, TMP-SMZ × 12 m	Recovered
14 (PR)	8/M	Consumption of raw goat’s milk	Spleen	1 : 640	*B. melitensis* (Blood, splenic aspirate)	Doxy × 6 weeks Inj. Strepto × 2 weeks	Recovered

NOTE: RUQ – right upper quadrant; Tet – tetracycline; m – months; LUQ – left upper quadrant; NS – not specified; Rif – rifampicin; TMP-SMZ – trimethoprimsulfamethoxazole; Doxy – doxycycline; Strepto – streptomycin; d – days; Genta – gentamicin; PR – present report

In conclusion, brucellosis in children may affect any organ system and imitate a variety of clinical entities. Clinicians practicing in endemic areas must be familiar with the varied and unusual presentations of this disease and include it in their differential diagnosis so that early diagnosis results in lower morbidity. Also, we stress the need for repeated serological evaluation for brucella using standardized antigens, especially in the background of epidemiological history and a clinical diagnosis.
